# Occupation and three-year incidence of respiratory symptoms and lung function decline: the ARIC Study

**DOI:** 10.1186/1465-9921-13-24

**Published:** 2012-03-20

**Authors:** Maria C Mirabelli, Stephanie J London, Luenda E Charles, Lisa A Pompeii, Lynne E Wagenknecht

**Affiliations:** 1 Department of Epidemiology and Prevention, Division of Public Health Sciences, Wake Forest School of Medicine, Winston-Salem, North Carolina, USA; 2 Epidemiology Branch, National Institute of Environmental Health Sciences, National Institutes of Health, Department of Health and Human Services, Research Triangle Park, North Carolina, USA; 3 Biostatistics and Epidemiology Branch, Health Effects Laboratory Division, National Institute for Occupational Safety and Health, Centers for Disease Control and Prevention, Department of Health and Human Services, Morgantown, West Virginia, USA; 4 Division of Epidemiology, Human Genetics and Environmental Sciences, The University of Texas School of Public Health, Houston, Texas, USA; 5 Division of Public Health Sciences, Wake Forest School of Medicine, Winston-Salem, North Carolina, USA

**Keywords:** ARIC study, epidemiology, occupation, respiratory tract disease

## Abstract

**Background:**

Specific occupations are associated with adverse respiratory health. Inhalation exposures encountered in these jobs may place workers at risk of new-onset respiratory disease.

**Methods:**

We analyzed data from 8,967 participants from the Atherosclerosis Risk in Communities (ARIC) study, a longitudinal cohort study. Participants included in this analysis were free of chronic cough and phlegm, wheezing, asthma, chronic bronchitis, emphysema, and other chronic lung conditions at the baseline examination, when they were aged 45-64 years. Using data collected in the baseline and first follow-up examination, we evaluated associations between occupation and the three-year incidence of cough, phlegm, wheezing, and airway obstruction and changes in forced expiratory volume in one second (FEV_1_) and forced vital capacity (FVC) measured by spirometry. All associations were adjusted for age, cigarettes per day, race, smoking status, and study center.

**Results:**

During the approximately three-year follow-up, the percentage of participants developing chronic cough was 3%; chronic phlegm, 3%; wheezing, 3%; and airway obstruction, defined as FEV_1 _< lower limit of normal (LLN) and FEV_1_/FVC < LLN, 2%. The average annual declines in FEV_1 _and FVC were 56 mL and 66 mL, respectively, among men and 40 mL and 52 mL, respectively, among women. Relative to a referent category of managerial and administrative support occupations, elevated risks of new-onset chronic cough and chronic phlegm were observed for mechanics and repairers (chronic cough: RR: 1.81, 95% CI: 1.02, 3.21; chronic phlegm: RR: 2.10, 95% CI: 1.23, 3.57) and cleaning and building service workers (chronic cough: RR: 1.85, 95% CI: 1.01, 3.37; chronic phlegm: RR: 2.28, 95% CI: 1.27, 4.08). Despite the elevated risk of new-onset symptoms, employment in cleaning and building services was associated with attenuated lung function decline, particularly among men, who averaged annual declines in FEV_1 _and FVC of 14 mL and 23 mL, respectively, less than the declines observed in the referent population.

**Conclusions:**

Employment in mechanic and repair jobs and cleaning and building service occupations are associated with increased incidence of respiratory symptoms. Specific occupations affect the respiratory health of adults without pre-existing respiratory health symptoms and conditions, though long-term health consequences of inhalation exposures in these jobs remain largely unexplored.

## Background

Exposure to inhalation hazards in the workplace can initiate respiratory symptoms among previously asymptomatic individuals [1,2], exacerbate symptoms among individuals with existing respiratory disease [3,4], and impair lung function [5-8]. Population-based epidemiologic research has identified specific occupations and workplace exposures that affect the prevalence and risk of respiratory outcomes [2,9-12]. One such study has suggested the influence of a healthy hire effect, as indicated by inverse associations observed between asthma onset before entering the workforce and subsequent employment in jobs involving inhalation exposures known or suspected to affect respiratory health [13].

Among young adults without asthma, prospective cohort data from general population-based research have shown elevated risks of new-onset asthma among men and women in nursing and cleaning professions and those whose jobs involved exposures to high molecular weight agents such as latex and flour [2,14-16]. Data available from the Atherosclerosis Risk in Communities (ARIC) study provide an opportunity to extend our understanding of occupational risks for the new-onset of respiratory symptoms and excess lung function decline in the general population by investigating such risks in a population-based cohort of adults. Specifically, we designed this analysis to evaluate the three-year cumulative incidence of self-reported chronic cough, chronic phlegm, current wheezing, and changes in lung function in a population of adults in the United States. These analyses extend previous findings of elevated prevalences of respiratory outcomes among men and women employed in cleaning, construction and extractive trades, mechanic and repair jobs, and transportation [17] by further evaluating the occupational risk of incident respiratory outcomes in these and other common occupations.

## Methods

### The ARIC study

We conducted an epidemiological analysis using data collected in the ARIC study, a prospective cohort study designed to assess the etiology of atherosclerosis and its clinical sequelae in a general population-based sample of men and women, aged 45-64 years, enrolled from four communities: Forsyth County, North Carolina; Jackson, Mississippi; the suburbs of Minneapolis, Minnesota; and Washington County, Maryland. Survey of the ARIC study population began with a baseline examination ('visit 1') that included detailed questionnaire and clinical evaluations. Approximately three years later, members of the cohort returned for a follow-up examination ('visit 2'). At both visits, participants completed spirometry and interviewer-administered questionnaires to provide information about his/her health history, current health status, and other related factors. Institutional review boards of participating study centers approved the study protocol and instruments and participants provided written informed consent. A detailed description of the ARIC study design and methods are available elsewhere [18].

### Final study population

Of the 15,792 participants who completed the visit 1 exam, we identified 8,967 participants who completed the visit 2 exam; had complete data for the variables included in our final models; did not report chronic cough, chronic phlegm, wheezing symptoms, or diagnoses of asthma, chronic bronchitis, emphysema, or other chronic lung conditions in the visit 1 exam; were not categorized as having airway obstruction based on spirometry completed in the visit 1 exam; and for whom the best forced vital capacity (FVC) measurement at visit 2 was generated during an exhalation of at least six seconds (Table 1). As in previous analyses [17], we categorized participants as having airway obstruction based on having forced expiratory volume in one second (FEV_1_) and FEV_1_/FVC measurements lower than the lower limits of normal (LLN) [19]. The 474 participants with FEV_1 _< LLN and FEV_1_/FVC < LLN at visit 1 were categorized as having airway obstruction and were excluded from our analyses.

**Table 1:** Selection of the final study population: the ARIC study

	**No. Included**	**No. excluded**
	
ARIC study visit 1 participants	15,649^1^	
Completed visit 2 examination	14,218	
Excluded due to missing data		
Missing respiratory health data		780
Missing smoking status		172
Missing current or most recent occupation		12
Excluded due to self-reported respiratory symptoms^2 ^and conditions^3 ^at visit 1		3,289
Excluded due to airway obstruction^4 ^at visit 1		474
Excluded due to best visit 1 FVC measurement generated with an exhalation of < 6 seconds		524
Final study population	8,967	

^1^Excludes participants who did not consent to the use of their data for non-cardiovascular health research (n = 41), races other than black or white (n = 48), and black participants recruited from suburbs of Minneapolis, Minnesota or Washington County, Maryland (n = 54) excluded to avoid prohibitively small numbers of participants in these strata of race and study center.

^2^Chronic cough, chronic phlegm, or wheezing

^3^Asthma, chronic bronchitis, emphysema, or other chronic lung disease

^4^Defined as FEV_1 _< LLN and FEV_1_/FVC < LLN

### Occupational classification

At the visit 1 examination, each participant reported his/her current employment status and current or most recent occupation. Current employment status was used to identify homemakers; for the remaining participants, occupations identified as the current or most recent occupation were assigned a three-digit occupation code using the occupational classification system used for the 1980 Census of Population and Housing [20]. Coding was performed centrally at the ARIC Coordinating Center. As in previous analyses of ARIC study data [17], we grouped occupation codes into major categories of occupations using categories published by the 1980 Census of Population and Housing and within each major category we pooled occupational groups that included < 1% of the original study population (n < 152) into three groupings of "other" occupations (i.e., "other service occupations," "other precision occupations," "other machine operating occupations").

### Respiratory outcomes

New-onset respiratory symptoms considered in this analysis include chronic cough, chronic phlegm, and wheezing. Chronic cough was assessed using responses to a question about cough ("Do you usually cough as much as four to six times a day, four or more days out of the week?"). Chronic phlegm was assessed using a question that referred to bringing up phlegm ("Do you usually bring up phlegm as much as twice a day, four or more days out of the week?"). Wheezing was evaluated using response to a question about wheezing symptoms ("Does your chest ever sound wheezy or whistling apart from [when you have a cold]?"). Each of these survey questions used to evaluate new-onset chronic cough, chronic phlegm, and wheezing were identical in wording to the questionnaire items included at visit 1.

The spirometry methods used in the ARIC study are described in detail elsewhere [17,21,22]. Testing methods were standardized across the four field centers and quality control measures were coordinated by a single pulmonary function reading center [22]. Bronchodilation was not included in the spirometry protocol. Spirometry measurements used in this analysis are annual changes in FEV_1 _and FVC in mL, calculated as the difference between the best measurements collected at ARIC visit 2 minus the best measurements collected at visit 1, divided by the length of time, in years, between the two measurements. We categorized participants with FEV_1 _< LLN and FEV_1_/FVC < LLN at visit 2 as having new-onset airway obstruction.

Our analyses include age, height, race, and sex. Age was categorized into quartiles of the age distribution of our final study population at visit 1 (45-49, 50-54, 55-59, and 60-64). We used information collected at visit 2 to categorize each participant as a current smoker, former smoker with three or less years since last cigarette, former smoker with more than three years since last cigarette, or lifetime non-smoker. The categorization of former smokers as having smoked within the last three years was designed to identify individuals who had smoked since the visit 1 exam. Among current smokers, cigarette use was reported as the number of cigarettes smoked per day, on average; for current smokers who reported smoking less than one cigarette per day, former smokers, and lifetime non-smokers, the number of cigarettes smoked per day was set to zero.

### Statistical analysis

We evaluated the associations between occupational categories and the risk of chronic cough, chronic phlegm, and wheezing in separate models using Poisson regression, specified with a log link and robust error variance estimation. For each of the four outcomes, associations were examined using a single model in which the incidence of the outcome was generated in all occupational categories, relative to that in the referent category. The referent occupational category was comprised of individuals who reported managerial occupations or administrative support occupations, including clerical jobs. All models were adjusted for age, number of cigarettes per day, race, sex, smoking status, and study center. Associations are presented as relative risks (RRs) with 95% CIs, indicating the risk of new-onset symptoms among individuals in each occupational category to that of respondents in the referent category.

Associations between occupational categories and mean annual changes in FEV_1 _and FVC were evaluated using linear regression models, adjusted for age (as a continuous variable), age squared, height, height squared, number of cigarettes per day, race, smoking status, and study center. For each lung function measure, sex-specific effect estimates were each generated using a single model and are presented as differences in the lung function metric for each occupational groups compared to those values in the referent category. The estimates shown are changes in FEV_1 _and FVC in mL, with 95% CIs. Adjusted changes that are smaller in magnitude than those observed in the referent category are indicated by negative signs. All analyses were conducted using SAS version 9.2 (SAS Institute Inc., Cary, North Carolina, USA).

## Results

Twenty-eight percent (28%, N = 3,763) of participants eligible for inclusion in our analysis were excluded due to the presence of respiratory health symptoms or conditions at ARIC visit 1 (Table 1). Characteristics of this excluded population and the final study population (N = 8,967) are shown in Table 2. Overall, the excluded population included higher percentages of men (49% versus 44%), current smokers (39% versus 16%), and former smokers with three or less years since his/her last cigarette (7% versus 4%), compared to the final study population. In our final study population, the most commonly reported jobs were those of managerial and administrative support (28%), professional specialties (16%), technical and sales occupations (12%), and homemakers (9%) (Table 3). During the approximately three-year follow-up period between ARIC study visits 1 and 2, new-onset chronic cough, chronic phlegm, and wheezing were each reported by more than 3% of participants and new-onset airway obstruction was identified by spirometry in 2%. Despite the small numbers of participants in individual occupational categories, statistically elevated risks of chronic cough and chronic phlegm were observed for mechanics and repairers (chronic cough: RR: 1.81, 95% CI: 1.02, 3.21; chronic phlegm: RR: 2.10, 95% CI: 1.23, 3.57), and cleaning and building service workers (chronic cough: RR: 1.85, 95% CI: 1.01, 3.37; chronic phlegm: RR: 2.28, 95% CI: 1.27, 4.08). In addition to cleaning and building service, private household service (RR: 1.79, 95% CI: 1.07, 3.01), health service (RR: 2.58, 95% CI: 1.43, 4.68), and other service (RR: 2.59, 95% CI: 1.35, 4.95) occupations were also associated with elevated risks of chronic phlegm. The highest risk of wheezing (5.8%) was generated for food preparation and service occupations (RR: 1.88, 95% CI: 1.03, 3.41). Occupation was not associated with the three-year incidence of airway obstruction.

**Table 2:** Characteristics of the final study population and participants excluded due to pre-existing respiratory symptoms or conditions: the ARIC study

	Excluded participants^1 ^(N = 3,763)	Final study population (N = 8,967)	
		
	No. (%)	No. (%)	
Age, in years			
45-49	921 (24.5)	2,475 (27.6)	
50-54	937 (24.9)	2,424 (27.0)	
55-59	954 (25.4)	2,167 (24.2)	
60-65	951 (25.3)	1,901 (21.2)	
*χ*^2^_3df _test			*p *< 0.01
Race			
Black	742 (19.7)	2,089 (23.3)	
White	3,021 (80.3)	6,878 (76.7)	
*χ*^2^_1df _test			*p *< 0.01
Sex			
Female	1,930 (51.3)	5,018 (56.0)	
Male	1,833 (48.7)	3,949 (44.0)	
*χ*^2^_1df _test			*p *< 0.01
Smoking status			
Current smoker	1,479 (39.3)	1,460 (16.3)	
No. cigarettes per day, on average Mean ± SD	21.0 ± 12.5	15.3 ± 10.3	
Median	20.0	15.0	
Minimum-Maximum	< 1 - 80	< 1 - 80	
Wilcoxon-Mann-Whitney test			*p *< 0.01
Former smoker, ≤ 3 years since last cigarette	259 (6.9)	354 (3.9)	
Former smoker, > 3 years since last cigarette	1,071 (28.5)	3,253 (36.3)	
Lifetime non-smoker	954 (25.4)	3,900 (43.5)	
*χ*^2^_3df _test			*p *< 0.01

^1 ^Excluded due to self-reported respiratory health symptoms or conditions (n = 3,289) or airway obstruction (n = 474) at visit 1

**Table 3:** Associations between occupation and the three-year incidence of chronic cough, chronic phlegm, wheezing, and airway obstruction

	**Total**	**Chronic Cough**		**Chronic Phlegm**		**Wheezing**		**Airway Obstruction**	
	
	**No**.	**No. (%)**	**RR (95% CI)^1^**	**No. (%)**	**RR (95% CI)^1^**	**No. (%)**	**RR (95% CI)^1^**	**No. (%)**	**RR (95% CI)^1^**
	
**Total**	8,967	317 (3.5)		304 (3.4)		301 (3.4)		196 (2.2)	
**Managerial and Administrative Support Occupations**	2,474	70 (2.8)	1.00 (referent)	62 (2.5)	1.00 (referent)	74 (3.0)	1.00 (referent)	52 (2.1)	1.00 (referent)
**Professional Specialty Occupations**	1,407	29 (2.1)	0.73 (0.48, 1.13)	28 (2.0)	0.80 (0.51, 1.26)	36 (2.6)	0.93 (0.63, 1.38)	23 (1.6)	0.99 (0.61, 1.61)
**Technical and Sales Occupations**	1,035	25 (2.4)	0.88 (0.56, 1.38)	18 (1.7)	0.68 (0.41, 1.15)	31 (3.0)	1.01 (0.67, 1.53)	22 (2.1)	1.04 (0.64, 1.68)
**Service Occupations**									
Private household occupations	417	22 (5.3)	1.45 (0.89, 2.37)	20 (4.8)	1.79 (1.07, 3.01)	14 (3.4)	1.29 (0.72, 2.33)	9 (2.2)	1.24 (0.59, 2.60)
Protective service	90	2 (2.2)	0.87 (0.22, 3.50)	3 (3.3)	1.31 (0.42, 4.11)	3 (3.3)	1.13 (0.37, 3.43)	3 (3.3)	1.64 (0.53, 5.02)
Food preparation and service	226	7 (3.1)	0.85 (0.39, 1.87)	9 (4.0)	1.56 (0.78, 3.13)	13 (5.8)	1.88 (1.03, 3.41)	4 (1.8)	0.95 (0.34, 2.62)
Health service	202	9 (4.5)	1.23 (0.61, 2.50)	13 (6.4)	2.58 (1.43, 4.68)	7 (3.5)	1.17 (0.54, 2.53)	4 (2.0)	1.23 (0.45, 3.35)
Cleaning and building service	188	12 (6.4)	1.85 (1.01, 3.37)	13 (6.9)	2.28 (1.27, 4.08)	9 (4.8)	1.68 (0.85, 3.32)	1 (0.5)	0.33 (0.05, 2.42)
Other service occupations	164	6 (3.7)	1.16 (0.51, 2.67)	10 (6.1)	2.59 (1.35, 4.95)	7 (4.3)	1.50 (0.70, 3.22)	4 (2.4)	1.55 (0.56, 4.30)
**Farming, Forestry, and Fishing Occupations**	94	5 (5.3)	1.85 (0.76, 4.49)	2 (2.1)	0.76 (0.19, 3.11)	4 (4.3)	1.32 (0.49, 3.56)	3 (3.2)	1.51 (0.49, 4.69)
**Precision Occupations**									
Mechanics and repairers	250	14 (5.6)	1.81 (1.02, 3.21)	17 (6.8)	2.10 (1.23, 3.57)	7 (2.8)	0.81 (0.37, 1.77)	8 (3.2)	1.26 (0.59, 2.66)
Construction and extractive trades	282	13 (4.6)	1.62 (0.90, 2.91)	15 (5.3)	1.70 (0.97, 2.97)	9 (3.2)	1.02 (0.52, 2.03)	8 (2.8)	1.33 (0.63, 2.80)
Other precision occupations	297	15 (5.1)	1.55 (0.91, 2.66)	17 (5.7)	1.89 (1.13, 3.16)	13 (4.4)	1.26 (0.70, 2.30)	7 (2.4)	0.93 (0.44, 1.99)
**Machine Operating Occupations**									
Textile, apparel, furnishing machine operators	93	7 (7.5)	2.62 (1.20, 5.72)	7 (7.5)	3.72 (1.75, 7.91)	3 (3.2)	1.07 (0.34, 3.40)	1 (1.1)	0.68 (0.10, 4.79)
Machine operators, assorted materials	192	10 (5.2)	1.66 (0.88, 3.14)	9 (4.7)	1.71 (0.87, 3.36)	7 (3.6)	1.13 (0.53, 2.38)	8 (4.2)	2.02 (0.96, 4.23)
Hand working occupations	93	3 (3.2)	1.09 (0.35, 3.39)	6 (6.5)	2.63 (1.16, 6.00)	2 (2.2)	0.72 (0.18, 2.85)	3 (3.2)	1.68 (0.52, 5.39)
Motor vehicle operation	221	14 (6.3)	1.91 (1.07, 3.41)	12 (5.4)	1.61 (0.87, 2.97)	11 (5.0)	1.45 (0.76, 2.74)	8 (3.6)	1.53 (0.72, 3.27)
Transportation, excl. motor vehicle	83	4 (4.8)	1.63 (0.61, 4.35)	3 (3.6)	1.10 (0.35, 3.43)	4 (4.8)	1.42 (0.52, 3.83)	4 (4.8)	1.89 (0.70, 5.14)
Handlers, equipment cleaners, helpers, laborers	171	8 (4.7)	1.46 (0.71, 3.00)	9 (5.3)	1.89 (0.95, 3.73)	9 (5.3)	1.70 (0.86, 3.34)	6 (3.5)	1.69 (0.75, 3.85)
Other machine operating occupations	164	8 (4.9)	1.62 (0.80, 3.27)	9 (5.5)	1.95 (0.97, 3.94)	6 (3.7)	1.14 (0.50, 2.57)	2 (1.2)	0.58 (0.14, 2.32)
**Homemaker, no other job reported**	824	34 (4.1)	1.51 (0.99, 2.31)	22 (2.7)	1.57 (0.94, 2.60)	32 (3.9)	1.26 (0.81, 1.94)	16 (1.9)	1.06 (0.59, 1.88)

^1 ^Adjusted for age, number of cigarettes per day, race, sex, smoking status, and study center

Table 4 shows associations between occupation and the average change in lung function measurements per year by sex. Among men, FEV_1 _and FVC declined an average of 56 mL (standard deviation: 69 mL) and 66 mL (standard deviation: 89 mL), respectively, per year. Among women, FEV_1 _and FVC declined an average of 40 mL (standard deviation: 50 mL) and 52 mL (standard deviation: 68 mL), respectively, per year. We observed statistically significantly greater declines in FEV_1 _among men in handworking occupations (-22.34 mL; 95% CI: -43.58, -1.18) and in FVC among men in food preparation and service occupations (-37.99 mL; 95% CI: -74.37, -1.60) than in the referent population of participants with managerial and administrative support jobs. In contrast, declines in these indices were attenuated among men and women working in cleaning and building services, compared with the referent population. Among women, none of the changes in FEV_1 _or FVC reached statistical significance.

**Table 4:** Associations between occupation and adjusted mean annual change in FEV_1 _and FVC

	**Men** **(N = 3,949)**			**Women** **(N = 5,018)**		
	
	**No. (%)**	**FEV_1_, in mL^1^**	**FVC, in mL^1^**	**No. (%)**	**FEV_1_, in mL^1^**	**FVC, in mL^1^**
	
**Managerial and Administrative Support Occupations**	1,082 (27.4)	0.00 (referent)	0.00 (referent)	1,392 (27.7)	0.00 (referent)	0.00 (referent)
**Professional Specialty Occupations**	633 (16.0)	-2.66 (-9.33, 4.01)	1.69 (-6.88, 10.25)	774 (15.4)	0.64 (-3.82, 5.09)	-1.68 (-7.78, 4.41)
**Technical and Sales Occupations Service Occupations**	577 (14.6)	-1.50 (-8.36, 5.37)	-1.69 (-10.51, 7.12)	458 (9.1)	-0.86 (-6.11, 4.40)	-7.08 (-14.26, 0.11)
Private household occupations	2 (0.1)	-14.80 (-108.99, 79.39)	-45.21 (-166.1, 75.68)	415 (8.3)	-2.67 (-8.31, 2.98)	-2.92 (-10.64, 4.80)
Protective service	68 (1.7)	7.02 (-9.71, 23.74)	16.29 (-5.18, 37.76)	22 (0.4)	18.32 (-2.60, 39.24)	9.8 (-18.83, 38.43)
Food preparation and service	23 (0.6)	-25.04 (-53.39, 3.31)	-37.99 (-74.37, -1.60)	203 (4.1)	1.08 (-6.36, 8.53)	-1.39 (-11.58, 8.80)
Health service	13 (0.3)	-16.61 (-53.99, 20.77)	-11.24 (-59.22, 36.74)	189 (3.8)	1.37 (-6.35, 9.09)	-0.79 (-11.35, 9.77)
Cleaning and building service	89 (2.3)	14.43 (-0.51, 29.36)	22.96 (3.79, 42.12)	99 (2.0)	4.34 (-5.94, 14.61)	8.33 (-5.74, 22.39)
Other service occupations	27 (0.7)	16.64 (-9.39, 42.68)	12.39 (-21.03, 45.80)	137 (2.7)	0.04 (-8.71, 8.80)	1.44 (-10.54, 13.42)
**Farming, Forestry, and Fishing Occupations**	79 (2.0)	8.54 (-7.14, 24.23)	-0.34 (-20.47, 19.79)	15 (0.3)	16.35 (-8.91, 41.61)	15.65 (-18.92, 50.22)
**Precision Occupations**						
Mechanics and repairers	238 (6.0)	-2.11 (-11.68, 7.47)	1.61 (-10.69, 13.90)	12 (0.2)	-2.72 (-30.95, 25.52)	8.44 (-30.21, 47.08)
Construction and extractive trades	275 (7.0)	0.62 (-8.43, 9.66)	-3.41 (-15.02, 8.20)	7 (0.1)	23.08 (-13.82, 59.99)	-20.08 (-70.59, 30.42)
Other precision occupations	219 (5.6)	-7.16 (-17.06, 2.74)	-4.69 (-17.39, 8.02)	78 (1.6)	4.71 (-6.63, 16.06)	12.15 (-3.37, 27.68)
**Machine Operating Occupations**						
Textile, apparel, furnishing machine operators	8 (0.2)	-25.17 (-72.50, 22.17)	-75.31 (-136.06, -14.55)	85 (1.7)	5.37 (-5.58, 16.32)	0.08 (-14.91, 15.06)
Machine operators, assorted materials	101 (2.6)	6.73 (-7.28, 20.75)	8.76 (-9.23, 26.75)	91 (1.8)	-9.47 (-20.05, 1.10)	-8.70 (-23.18, 5.77)
Hand working occupations	41 (1.0)	-22.38 (-43.58, -1.18)	-26.11 (-53.32, 1.10)	52 (1.0)	-3.92 (-17.67, 9.83)	-9.78 (-28.60, 9.05)
Motor vehicle operation	196 (5.0)	-1.40 (-11.88, 9.08)	-6.14 (-19.59, 7.32)	25 (0.5)	4.98 (-14.72, 24.68)	-0.17 (-27.13, 26.79)
Transportation, excl. motor vehicle	78 (2.0)	-2.66 (-18.33, 13.00)	3.11 (-17.00, 23.21)	5 (0.1)	18.38 (-25.38, 62.13)	13.78 (-46.11, 73.67)
Handlers, equipment cleaners, helpers, laborers	109 (2.8)	-3.19 (-16.7, 10.32)	-1.18 (-18.52, 16.16)	62 (1.2)	-5.93 (-18.56, 6.70)	-2.10 (-19.39, 15.19)
Other machine operating occupations	85 (2.2)	4.22 (-10.86, 19.30)	7.8 (-11.56, 27.16)	79 (1.6)	-1.43 (-12.75, 9.89)	-12.44 (-27.93, 3.05)
**Homemaker, no other job reported**	6 (0.2)	-25.84 (-80.58, 28.90)	28.89 (-41.37, 99.16)	818 (16.3)	0.08 (-4.43, 4.59)	0.71 (-5.47, 6.88)

^1^Mean change (with 95% CI) per year, adjusted for age, age squared, height, height squared, number of cigarettes per day, race, smoking status, and study center

## Discussion

Our study evaluated the cumulative incidence of respiratory symptoms and average annual changes in FEV_1 _and FVC over an approximately three-year follow-up period between the baseline ARIC study examination (1987-1989) and the first follow-up (1990-1992). We observed elevated risk of new-onset respiratory symptoms among ARIC study participants who reported working in specific occupations, including mechanic and repair occupations and cleaning and building services. These results extend previous findings of elevated prevalences of asthma, chronic bronchitis, and chronic cough among ARIC study participants employed in precision production and service occupations [17]. Together with these previous findings, our data highlight occupations in which men and women may experience inhalation exposures that increase their risk of both exacerbation of existing respiratory disease and initiation of new symptoms.

Previous research into the incidence of asthma in a general population-based sample has suggested that workplace exposures cause more than 10% of all adult-onset asthma [2]. Men and women working in nursing and cleaning jobs and those with exposures to cleaning products or high molecular weight agents such as animal antigens, enzymes, and natural rubber latex have been identified as at elevated risk [2], as have men and women employed in agriculture [23], manufacturing [23], and welding [24]. Our findings of elevated risk of adult-onset respiratory symptoms among mechanics and repairers and cleaning and building service workers are consistent with existing information about the initiation of respiratory disease by agents encountered on the job [1]. Additional research designed to identify the specific agents and mixtures of those agents and to describe the workplace activities and behaviors that may be responsible for the observed associations may provide useful information with which to target workplace interventions and monitoring efforts. Workplace interventions, such as those reducing natural rubber latex exposure and sensitization among health care workers [25], may be effective in reducing exposures to respiratory irritants and sensitizers in other workplaces. Monitoring the respiratory health of workers in high-risk occupations would provide valuable information about the role of long-term occupational exposures in the progression of lung disease.

Despite the elevated risks of new-onset respiratory symptoms in these populations, we did not observe statistically significant elevations in the risk of airway obstruction or lung function decline. *A priori *we excluded from our analysis of lung function decline 2,422 participants with self-reported respiratory symptoms or conditions at visit 1, but for whom FEV_1 _≥ LLN or FEV_1_/FVC ≥ LLN - that is, individuals who were symptomatic, but who did not meet our definition of airway obstruction. Inclusion of these excluded individuals to our analyses of lung function decline did not notably affect our results (not shown). In symptomatic population, the annual declines in FEV_1 _(men: -60 mL; women: -43 mL) and FVC (men: -69 mL; women: -55 mL) were larger than those observed in the final study population, suggesting that self-reported symptoms may indicate future lung function decline. We also *a priori *excluded from our analysis 524 study participants for whom the best FVC measurement was obtained during an exhalation of fewer than six seconds. If occupation is associated with lung function decline and lung function decline is associated with the inability to exhale for six seconds, than our analyses may not include participants most severely affected by workplace inhalation exposures and our results may underestimate the true association between occupation and new-onset respiratory disease. Among these 524 excluded participants, the three-year cumulative incidences of chronic cough (5.0%), chronic phlegm (3.6%), and wheezing (4.0%) were each higher than those observed among the final, included, study population. However, none of the excluded participants in the two occupation groups identified at highest risk (i.e., mechanic and repair occupations, cleaning and building services) for new-onset symptoms reported the conditions and these two populations were not disproportionately represented in the excluded population, suggesting that this exclusion criterion did not reduce the external validity of our study. Indeed, our findings of elevated respiratory symptom incidences in these occupations suggest that these workers may be at particular risk for the development of more severe respiratory disease and should be monitored for these and other long-term respiratory health outcomes.

The limitations of using these data to evaluate associations between occupation and respiratory health have been described previously [17]. The results presented here do not take into account many of the behavioral, medical, or psychosocial factors that may be associated with both occupation and respiratory health. Because of the small number of participants in each occupational category with incident respiratory symptoms, we were unable to evaluate potential joint effects (i.e., interactions) of tobacco use and occupation in these data; however, the role of tobacco use in the associations between occupation and respiratory health outcomes was effectively accounted for in our analyses by the inclusion of smoking history information provided at visit 2. Using these data, we evaluated changes in lung function during an approximately three year follow-up period. If lung function decline is associated with occupation, then observing changes in lung function during a longer period of follow-up may have generated not only larger absolute differences in lung function, but larger difference relative to the referent population. Furthermore, in these data, we observed a slightly higher cumulative incidence of wheezing than of chronic cough, chronic phlegm, or airway obstruction. If self-reported wheezing precedes chronic cough, chronic phlegm, airway obstruction, or clinically important declines in lung function, then occupations for which we observed higher incidences of wheezing may indicate those for which increased attention to controlling exposures to vapors, gases, dusts, and fumes in the workplaces has the potential to reduce long-term respiratory health effects among exposed workers. Neither detailed respiratory questionnaire items nor spirometry were included in ARIC study visits 3 or 4 examinations, thus our analyses are limited to evaluating three-year changes in these respiratory health outcomes. In this analysis, the combination of questionnaire-based information and measurements of lung function in a large, well-characterized, population-based cohort of adult men and women is a notable strength of our study.

In the ARIC study, occupations were categorized at visit 1 and risks identified here are attributed to these occupations without additional information about length of employment, job tasks, use of respiratory protection, or changes in employment during the follow-up period. Without information about these and other potentially important occupational factors, our classification of participants based on their self-reported occupation undoubtedly compounds misclassification of participants' occupations with exposure variations within each category. Our categorization of occupations also does not account for specific occupational exposures among individuals working in each occupation. In large population-based cohorts studies in which individual exposure assessment is not feasible, job exposure matrices (JEMs) have been used to assign specific asthmagenic exposures to individual occupations [26]. In the ARIC study, the application of an asthma-specific JEM could improve the classification of individuals with exposures known or suspected of initiating or exacerbating respiratory health. In these analyses, we retained the classification system based on occupation in order to draw conclusions about specific occupations; however, further analyses of these data may consider the incorporation of a JEM as an alternative occupational classification. That our study provides evidence of the potential respiratory health impact of specific high-risk occupations despite these limitations suggests that improvements in the quality of occupational exposure information would likely yield higher estimates of the associations between specific occupations and incident respiratory outcomes.

## Conclusions

Men and women in specific occupations may be at risk of developing work-related respiratory disease. The long-term respiratory health consequences of inhalation exposures among mechanics, repairers, cleaners and janitors, building service professionals, and other workers remain largely unexplored.

## Abbreviations

95% CI: 95 percent confidence interval; ARIC: Atherosclerosis Risk in Communities Study; FEV_1_: Forced expiration in one second; FVC: Forced vital capacity; LLN: Lower limit of normal; RR: Risk ratio; SD: Standard deviation

## Competing interests

The authors declare that they have no competing interests.

## Authors' contributions

MCM participated in the conception and design of the analysis, performed the statistical analysis, interpreted the data, and drafted the manuscript. SJL, LEC, and LAP participated in the design of the analysis, interpreted the data, and critically reviewed drafts of the manuscript. LEW participated in the conception and design of the analysis, interpreted the data, and critically reviewed drafts of the manuscript. All authors read and approved the final manuscript.

## Disclosure of funding

The Atherosclerosis Risk in Communities Study is carried out as a collaborative study supported by National Heart, Lung, and Blood Institute contracts (HHSN268201100005C, HHSN268201100006C, HHSN268201100007C, HHSN268201100008C, HHSN268201100009C, HHSN268201100010C, HHSN268201100011C, and HHSN268201100012C). Dr. London is supported by the Division of Intramural Research, National Institute of Environmental Health Sciences.
